# Evaluating the Efficacy of AI-Based Interactive Assessments Using Large Language Models for Depression Screening: Development and Usability Study

**DOI:** 10.2196/78401

**Published:** 2026-01-13

**Authors:** Zheng Jin, Jiaxing Hu, Dandan Bi, Kaibin Zhao, Huan Yu

**Affiliations:** 1 International Joint Laboratory of Behavior and Cognitive Science Zhengzhou Normal University Zhengzhou, Henan China; 2 School of Electrical, Computer and Telecommunications Engineering University of Wollongong Wollongong Australia; 3 Department of Psychology Zhejiang Normal University Jinhua, Zhejiang China

**Keywords:** large language models, automated assessment paradigm, AAP, natural language processing, NLP, ChatGPT, Beck Depression Inventory Fast Screen, BDI-FS, mental health assessment, artificial intelligence, AI, psychology

## Abstract

**Background:**

The evolution of language models, particularly large language models, has introduced transformative potential for psychological assessment, challenging traditional rating scale methods that have dominated clinical practice for over a century.

**Objective:**

This study aimed to develop and validate an automated assessment paradigm that integrates natural language processing with conventional measurement tools to assess depressive symptoms, exploring its feasibility as a novel approach in psychological evaluation.

**Methods:**

A cohort of 115 participants, including 28 (24.3%) individuals diagnosed with depression, completed the Beck Depression Inventory Fast Screen via a custom ChatGPT interface (BDI-FS-GPT) and the Chinese version of the Patient Health Questionnaire–9 (PHQ-9). Statistical analyses included the Spearman correlation (PHQ-9 vs BDI-FS-GPT scores), Cohen κ (diagnostic agreement), and area under the curve (AUC) evaluation.

**Results:**

Spearman analysis revealed a moderate correlation between PHQ-9 and BDI-FS-GPT scores. The Cohen κ indicated moderate diagnostic agreement between the PHQ-9 and the BDI-FS-GPT (κ=0.43; 76.5% agreement), substantial agreement between the BDI-FS-GPT and the clinical diagnosis (κ=0.72; 88.7% agreement), and moderate agreement between the PHQ-9 and the clinical diagnosis (κ=0.55; 71.4% agreement). The BDI-FS-GPT demonstrated excellent diagnostic accuracy (AUC=0.953) at a cutoff of 3, detecting 89.3% of participants with depression with an 11.5% false-positive rate compared to the PHQ-9 (AUC=0.859) at a cutoff of 5 (sensitivity=71.4%; false-positive rate=13.8%). Participants also reported significantly higher satisfaction with the automated assessment compared to the traditional scale (*P*=.02).

**Conclusions:**

The automated assessment paradigm framework combines the interactivity and personalization of natural language processing–powered tools with the psychometric rigor of traditional scales, suggesting a preliminary feasibility paradigm for future psychological assessment. Its ability to enhance engagement while maintaining reliability and validity provides encouraging evidence, warranting validation in larger and more diverse studies as large language model technology advances.

**International Registered Report Identifier (IRRID):**

RR2-10.1101/2024.07.19.24310543

## Introduction

Numerous studies have used numerical rating scales to capture participants’ feelings using predefined scoring responses to represent complex psychological states. When asked certain questions (eg, “Are you satisfied with your partner?”), people often provide descriptive, open-ended responses in words (eg, “Most of my expectations are met, but...”) rather than closed numerical or categorical answers (eg, 7=“strongly agree” or 1=“strongly disagree”). For over a century, rating scales have dominated psychological assessment, providing valuable insights but also limiting how people express more nuanced or atypical mental states [1-3]. As a result, important contextual information, such as how individuals interpret questions or which situational factors shape their responses, often remains unmeasured.

Natural language is our inherent way of conveying inner experiences and psychological states, characterized by high ecological validity and rich, multidimensional information [4]. Natural language models have advanced from early statistical methods (eg, n-grams) to neural architectures (eg, transformer), culminating in large language models (LLMs) trained with instruction tuning and reinforcement learning from human feedback. This advance enables the structured analysis of open-ended narratives, helping address the limitations of conventional rating scales [5,6]. Previous studies have validated the potential of natural language processing–based approaches in psychological measurement. Kjell et al [7,8], for example, demonstrated that latent semantic analysis and context-based embedding models such as bidirectional encoder representations from transformers can reliably quantify open-text responses, yielding results highly consistent with those of traditional psychometric scales. Similarly, Son et al [9] applied language analysis to early interview transcripts of 911 responders and successfully predicted the development of posttraumatic stress disorder symptoms, identifying specific linguistic markers such as word frequency patterns and topic distributions generated via latent Dirichlet allocation. These findings laid the groundwork for integrating natural language processing into psychological assessment but were largely limited to post hoc analyses of isolated text samples or sentiment classification.

Recent reviews have mapped the growing landscape of LLM applications in mental health, including depression detection, suicide risk prediction, and clinical decision support [10-13]. Representative studies illustrate current directions. Guo et al [14] built a GPT-3.5–based Patient Health Questionnaire–9 (PHQ-9) chatbot that guides users through fixed response options, returns a summed score, and provides resource information; the system was tested using simulated personas and a small convenience sample recruited online. Mixed methods work comparing chatbot responses with those of licensed therapists has described strengths such as validation and psychoeducation alongside safety gaps in crisis handling and inquiry depth [15]. Method proposals such as MAQuA and PsyLLM explore adaptive question selection and therapy navigation using LLMs [16,17].

Taken together, most prior systems rely on static, prelabeled text or fixed-option questionnaires (eg, Reddit, X [formerly known as Twitter], the DAIC-WOZ database, and Weibo); yield categorical labels or simple sum scores; and seldom map open-text responses to standardized, scale-anchored psychometric scores in real time. To our knowledge, few studies have evaluated such scores against a concurrent clinical diagnosis in a routine care setting. This study introduced the automated assessment paradigm (AAP), which embeds a validated rating scale (the Beck Depression Inventory Fast Screen [BDI-FS]) into an LLM-powered dialogue. This approach enables the automatic generation of standardized scores directly from participants’ natural language responses, bridging the flexibility of conversational artificial intelligence (AI) with the rigor and comparability of traditional psychometric tools.

Beyond methodological concerns, the application of AI to mental health raises critical ethical challenges. Mental health data are highly sensitive, and issues of privacy, data security, algorithmic bias, and potential misuse mean that technical validity alone is not sufficient. Equally important is whether individuals feel respected, comfortable, and willing to engage with AI-based systems when discussing their psychological states. Without participant trust and acceptance, even technically sound tools may fail in practice. Therefore, this study sought not only to test the diagnostic performance of an AAP that embeds a validated rating scale within an LLM-driven dialogue but also to examine whether such an approach is acceptable and engaging to users. By considering both measurement validity and participant experience, we aimed to provide a more balanced evaluation of the feasibility of AI-assisted psychological assessment. Given that text-based LLM assessment requires basic digital literacy and stable internet access, this study was conducted in a university-affiliated center that routinely serves urban adults, including both university students and local residents.

## Methods

### Participants

**Figure 1:**
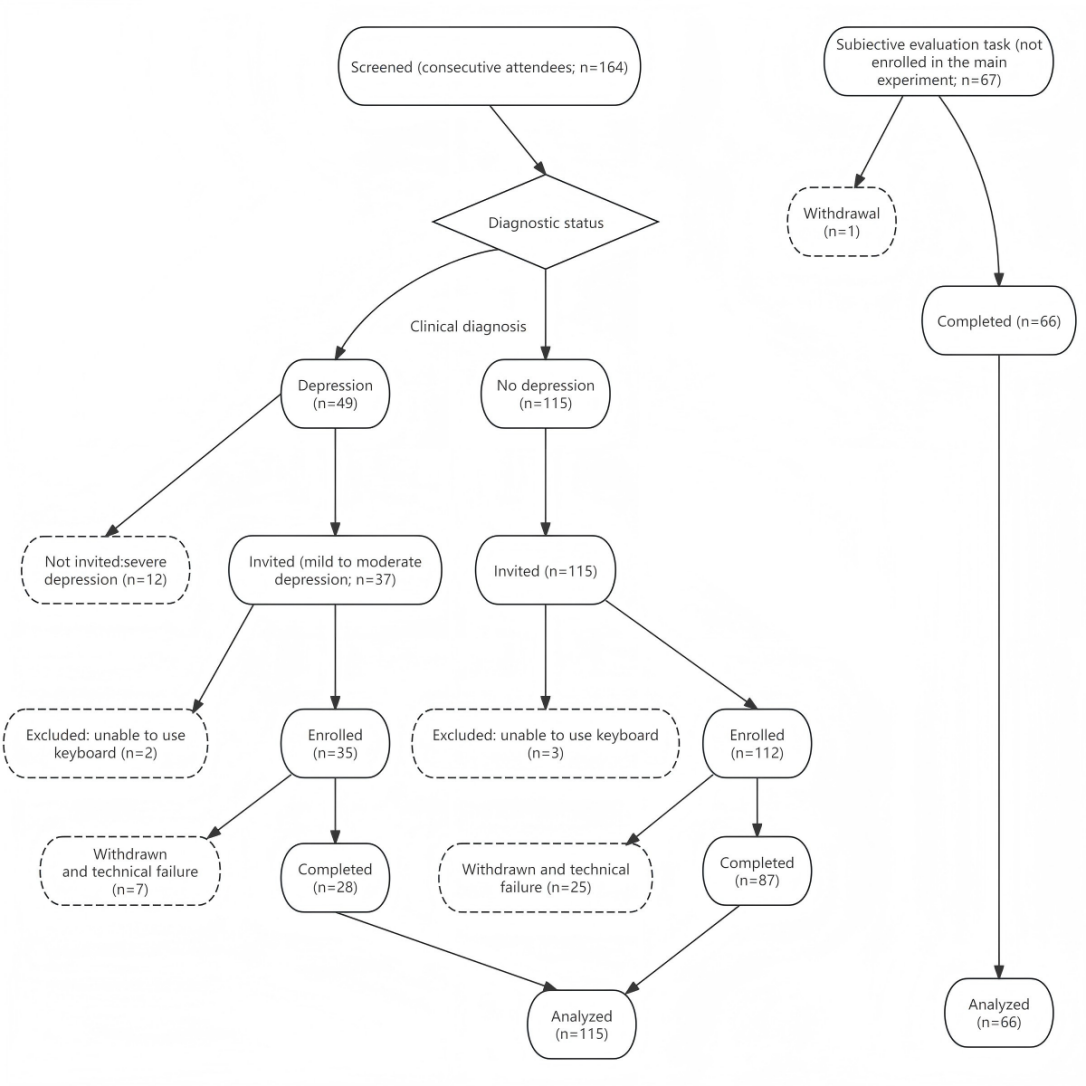
Participant flow from screening to analysis.

Participants were recruited from the Psychological Crisis Early Warning and Monitoring Technology Research Center at Zhengzhou Normal University in Zhengzhou, China, which provides routine psychological consultation and diagnosis for local residents and university students. Between July 2024 and January 2025, a total of 164 attendees (aged ≥18 years) were screened sequentially in the order they presented, comprising adults who provided consent, returned for the study session, and were classified as either diagnosed with depression or not diagnosed with depression, without any other primary mental disorder. Of these 164 attendees, 49 (29.9%) were diagnosed with depression, and 115 (70.1%) were not. All 115 individuals without depression were invited. Of the 49 individuals diagnosed with depression, 12 (24.5%) with severe depression were not invited to participate. The AAP in its current form is an exploratory tool and not yet validated for high-risk situations, so administering it to individuals who are severely ill without established safety evidence was considered ethically inappropriate. Of the 37 attendees in the depression group who were invited, 2 (5.4%) were excluded due to impairments that prevented completion of the assessment (ie, inability to interact with the keyboard), and 7 (18.9%) withdrew or could not complete the session because of technical interruptions (eg, sudden software errors or network failures), leaving 28 participants. In the group without depression, of the 115 attendees, 3 (2.6%) were excluded for testing-impairing conditions, and 25 (21.7%) withdrew, leaving 87 participants (Figure 1). Each diagnosis was conducted by a licensed psychiatrist on duty at the center according to the Diagnosis and Treatment of Mental Disorders Guidelines (2020 Edition) of the National Health Commission of the People’s Republic of China. A total of 67 additional university students participated in the satisfaction measurement regarding the assessment methods.

In the study by Elben et al [18], the BDI-FS demonstrated a significantly higher agreement with the clinician’s diagnosis of depression at the index visit than would be expected by chance, with a Cohen κ of 0.39 (81% agreement; *P*<.002). In contrast, Golden et al [19] reported that the BDI-FS showed a Cohen κ of 0.42 in agreement with the *Diagnostic and Statistical Manual of Mental Disorders, Fourth Edition* (*DSM-IV*) diagnosis, which they considered to be less than ideal. When setting the minimum acceptable κ (κ_0_) value to 0.39 and the expected κ (κ_1_) value to 0.8, with an estimated depression prevalence of approximately 30% among individuals seeking psychological consultation [20], a sample size of 85 participants was required to achieve a statistical power of 80% (1 – β) at a significance level of .05 [21]. The final analytic sample exceeded the minimum required sample size.

### Materials

The BDI-FS is an abbreviated version of the 21-item Beck Depression Inventory–II consisting of only 7 items [22]. It has been validated for use with the general population, college students, and inpatients [18,23], as well as in the Chinese population [24]. The diagnostic cutoff value for the BDI-FS is ≥4. On the basis of the BDI-FS, custom versions of ChatGPT-4o (OpenAI; BDI-FS-GPT) were created on June 7, 2024. Specifically, we implemented a fixed, prespecified sequence of 10 prompts based on the BDI-FS. For instance, when addressing self-criticalness in the BDI-FS, the prompt asks the respondent three questions: (1) “Compared to before, do you find yourself more self-critical?” (2) “Do you blame yourself for your shortcomings?” (3) “When something bad happens, do you worry it’s your fault?” The agent asks each question in order and waits for the participant’s response before proceeding. The agent provides only courteous responses without commenting on the answers. Scoring follows the original BDI-FS criteria. After collecting free-text responses, the agent assigns item scores of 0, 1, 2, or 3 by matching the aggregated meaning to the BDI-FS anchors (eg, self-blame: 0=“I do not blame myself,” 1=“I blame myself more than before,” 2=“I blame myself for my faults,” and 3=“I blame myself for anything bad that happens”). The aggregation and mapping steps are deterministic and rule based. Full prompts and deidentified response examples are available in our Open Science Framework repository [25]. A board-certified psychiatrist reviewed the exact Chinese prompts and the domain mapping before data collection to ensure semantic consistency with the BDI-FS and confirm the absence of potentially offensive wording.

The Patient Health Questionnaire, developed by Kroenke et al [26] based on the *DSM-IV*, is a self-assessment tool for evaluating mental disorders in primary health care settings. The PHQ-9 is a subset of the Patient Health Questionnaire used specifically to assess depression, consisting of 9 items based on the 9 symptom criteria for major depressive disorder as outlined in the *DSM-IV*. Regarding severity, the PHQ-9 comprises 5 categories, where a cutoff point of 0 to 4 indicates no depressive symptoms, 5 to 9 indicates mild depressive symptoms, 10 to 14 indicates moderate depressive symptoms, 15 to 19 indicates moderately severe depressive symptoms, and 20 to 27 indicates severe depressive symptoms.

The subjective evaluation was adapted from the Usefulness, Satisfaction, and Ease of Use Questionnaire [27] and included 5 items assessing perceived usefulness, considerateness, respect, satisfaction with the time required, and overall satisfaction. These measure satisfaction with the information obtained, the degree to which the process felt considerate, and the extent to which the measurement method made the respondent feel respected. The scale includes 3 reverse-scored items, with higher scores indicating greater comfort. The internal consistency of the acceptance measurement for the BDI-FS and BDI-FS-GPT was 0.67 and 0.72, respectively.

### Procedure

The center’s social-psychological services regularly accommodate inhabitants from the local communities and universities, who typically receive psychological consultation and diagnostic services. After obtaining participants’ consent, the research procedures were integrated into the regular intake process, with participants invited for a follow-up visit within 48 hours after the standard service. Participants completed the BDI-FS-GPT and the Chinese version of the PHQ-9 in a private space guided by 2 trained research assistants familiar with the operational procedures who were unaware of the diagnostic results. The order of the 2 assessments was counterbalanced, with a 2-minute interval between them. The assessment concluded when ChatGPT displayed the following concluding statements and final question: “Our conversation ends here. Thank you for your participation. Would you like to see your scores?” Participants then exited the program. The research assistant reviewed and recorded the scores. Risk management procedures were in place throughout. Individuals with severe depression or acute suicide risk as determined in the initial clinical assessment were not invited into the study and, instead, received appropriate clinical management and referral. If a participant’s responses indicated marked emotional distress (eg, suicidal ideation) during the AAP session, the research assistant immediately informed the on-duty psychiatrist, who conducted an in-person risk assessment and arranged appropriate follow-up in accordance with institutional protocols. The 67 participants in the satisfaction evaluation group did not participate in the aforementioned experiment. They only completed the traditional BDI-FS and BDI-FS-GPT measurement in a counterbalanced order and subsequently filled out the subjective evaluation scale, and 1.5% (1/67) withdrew from the study.

### Ethical Considerations

The experimental procedure was approved by the Academic Ethics Committee of Zhengzhou Normal University (approval ZZNU2023LL019). All participants provided written informed consent prior to participation. This included consent for the use of deidentified data in secondary analyses and for the publication of results. To protect participants’ privacy and confidentiality, only a limited set of demographic variables (sex, residence, and age) was retained in the final dataset. Participants were offered a ¥50 (US $7.10) incentive, although 13.9% (16/115) of the participants completed the procedure but declined the compensation. All participants were free to withdraw at any time without providing a reason.

## Results

Participant characteristics by depression status are summarized in Table 1. The Cronbach α was 0.80 (95% CI 0.725-0.847) for the PHQ-9 and 0.71 (95% CI 0.60-0.78) for the BDI-FS-GPT. Spearman correlation analysis showed significant correlation between the total scores on the PHQ-9 and the BDI-FS-GPT, with a correlation coefficient of 0.45 (*P*<.001). The agreement of diagnoses between the 2 measures as assessed using the Cohen κ was significant, with a value of 0.43 (76.5% agreement; *P*<.001). The BDI-FS-GPT diagnosis showed significantly higher agreement with the current diagnosis of depression, with a Cohen κ of 0.72 (88.7% agreement; *P*<.001). The PHQ-9 diagnosis also agreed significantly more often than chance with a present diagnosis of depression, as indicated by a significant Cohen κ of 0.55 (71.42% agreement; *P*<.001). Median PHQ-9 scores were 6 (IQR 4-8.75) in the depression group and 2 (IQR 0-4) in the nondepression group, and median BDI-FS-GPT scores were 6 (IQR 4-6.75) in the depression group and 0 (IQR 0-2) in the nondepression group.

**Table 1:** Participant characteristics by depression status (N=115).

			No depression		Depression
**Sex, n (%)**					
	Female	57/87 (65.5)		21/28 (75.0)	
	Male	30/87 (34.5)		7/28 (25.0)	
**Residence, n (%)**					
	Urban	66/87 (75.9)		23/28 (82.1)	
	Rural	21/87 (24.1)		5/28 (17.9)	
Age (years), mean (SD)			28.76 (8.84)		26.86 (7.25)
BDI-FS-GPT^a^ score (0-21), mean (SD)			0.75 (1.15)		5.36 (2.31)
PHQ-9^b^ score (0-27), mean (SD)			2.30 (2.13)		6.46 (3.55)

^a^BDI-FS-GPT: Beck Depression Inventory Fast Screen based on a custom ChatGPT interface.

^b^PHQ-9: Patient Health Questionnaire–9.

The area under the receiver operating characteristic (ROC) curve for the BDI-FS-GPT was 95.3% (95% CI 90.0%–100.0%). The ROC analysis indicated that a cutoff score of 3 provided the optimal balance between sensitivity (89.3%) and specificity (88.5%; Youden index=0.778), supporting its use as the primary threshold for the BDI-FS-GPT in this sample. For the PHQ-9, ROC analysis indicated that a cutoff of 5 provided the optimal balance between sensitivity (71.4%) and specificity (86.2%), with the highest Youden index (0.576). The BDI-FS-GPT demonstrated strong discrimination performance, with an area under the precision-recall curve of 0.921. For the sample in this study, the logistic regression model for the BDI-FS-GPT (cutoff=3; logit[p(depression)]=1.376BDI-FS-GPT – 4.741; *P*<.001) and the logistic regression model for the PHQ-9 (cutoff=5; logit[p(depression)]=0.574PHQ-9 – 3.438; *P*<.001) revealed better performance of the BDI-FS-GPT than the PHQ-9. Sensitivity, specificity, positive predictive value, and negative predictive value across alternative cutoff values for both instruments are presented in Table 2.

The confusion matrix for the BDI-FS-GPT classification (cutoff=3) against the clinical diagnosis is shown in Table 3.

**Table 2:** Sensitivity, specificity, positive predictive value (PPV), and negative predictive value (NPV) at different cutoffs for the Beck Depression Inventory Fast Screen based on a custom ChatGPT interface (BDI-FS-GPT) and Patient Health Questionnaire–9 (PHQ-9).

Cutoff value		Sensitivity	Specificity	PPV	NPV
**BDI-FS-GPT**					
	1	0.964	0.655	0.474	0.983
	2	0.964	0.736	0.54	0.985
	3	0.893	0.885	0.714	0.963
	4	0.786	0.977	0.917	0.934
	5	0.643	1	1	0.897
**PHQ-9**					
	1	0.964	0.299	0.307	0.963
	2	0.964	0.402	0.342	0.972
	3	0.929	0.575	0.413	0.962
	4	0.821	0.713	0.479	0.925
	5	0.714	0.862	0.625	0.904
	6	0.536	0.931	0.714	0.862
	7	0.393	0.954	0.733	0.83
	8	0.286	0.977	0.8	0.81
	9	0.25	0.989	0.875	0.804

**Table 3:** Confusion matrix for the Beck Depression Inventory Fast Screen based on a custom ChatGPT interface (BDI-FS-GPT) versus clinical diagnosis.

			Clinical depression		
			Positive (n=28)		Negative (n=87)
**BDI-FS-GPT**					
	Positive (n=35)	25 TPs^a^		10 FPs^b^	
	Negative (n=80)	3 FNs^c^		77 TNs^d^	

^a^TP: true positive.

^b^FP: false positive.

^c^FN: false negative.

^d^TN: true negative.

Participants reported slightly higher satisfaction with the AAP powered by generative pretrained transformer–based language models (mean 20.26, SD 2.46; range 13-25) compared to the traditional BDI-FS (mean 19.50, SD 2.60; range 11-24). The Wilcoxon signed rank test confirmed that this difference was statistically significant (*z*=−2.35; *P*=.02), indicating a preference for the more interactive, AI-driven assessment process.

## Discussion

The BDI-FS-GPT demonstrated acceptable internal consistency. The high correlation between the PHQ-9 and BDI-FS-GPT suggests that they are consistent in assessing depressive symptoms. This result strengthens our confidence in the congruence and validity of the BDI-FS-GPT compared to traditional scales measuring the same construct. The diagnostic agreement between both tools and actual diagnoses was significantly higher than random consistency. The BDI-FS-GPT showed particularly high agreement with the actual depression diagnosis. Specifically, a BDI-FS-GPT score of ≥3 is better interpreted as a flag for further clinical assessment rather than a definitive diagnosis, balancing a low miss rate with an acceptable number of false positives. Although discrimination performance was strong (area under the precision-recall curve=0.921), calibration analysis indicated that the model tended to underestimate depression risk on average (calibration intercept=−4.741) and produced a calibration slope greater than 1 (1.376), suggesting that symptom differences may be amplified at higher severity levels. These calibration characteristics are expected in early-phase models developed on modest sample sizes.

Typically, the development of rating scales follows a rigorous process of validity and reliability testing to establish scientific scoring rules and norms. The logic of the AAP is relatively simple: it adapts traditional scale questions into a structured interview format, which is then administered via an AI interactive question-and-answer system. The AI system collects the responses, and semantic analysis based on traditional scoring rules is used to automatically assign scores. While existing research has explored various integrations of AI, structured interviews, and rating scales [28-31], there are no direct examples to our knowledge that integrate these elements into a system and predict actual symptoms in practice. In some studies, AI has been used to assist with rating scales, such as sentiment analysis or emotion recognition based on text analysis, or assess participants’ emotional tendencies or mental states. For instance, AI has been used to classify emotions in responses to open-ended questions, but these studies have generally used predefined sentiment analysis models and not incorporated semantic automatic scoring of rating scales, often focusing on specific areas such as sentiment analysis or emotion recognition rather than comprehensive psychological measurement. Other studies have attempted to apply AI or machine learning to automate the scoring systems of traditional rating scales, primarily focusing on automating analysis and pattern recognition of survey data.

In contrast to traditional structured interviews, the virtual avatar-based assessment method significantly reduces human interference, addresses the privacy limitations of structured interviews, and saves time costs. With further advancements in AI technology, questions from classic rating scales could be presented in multimodal formats. The use of the AAP provides a more participant-centered, open-ended, and interactive experience for participants while retaining psychometric reliability, marking a new direction for digital mental health measurement. Because the BDI-FS-GPT is fully automated, a natural next step is to embed it as a previsit or waiting room screener in multiple clinics coupled with predefined referral or follow-up rules when scores exceed agreed thresholds. Future trials can use this workflow to evaluate longitudinal stability, responsiveness to change, and the impact on clinicians’ workload and triage decisions. While the AAP demonstrated satisfaction, the integration of conversational AI into mental health requires careful consideration of the user-agent relationship. Previous research by Brandtzæg et al [32] has highlighted that young users can form emotional attachments to chatbots used for social support, which poses risks if the AI fails to respond appropriately to distress. However, it is crucial to distinguish the AAP from companion-oriented chatbots. The AAP is designed as a brief, structured clinical screening tool rather than a platform for longitudinal emotional support. The interaction is goal oriented and confined to specific diagnostic inquiries, which inherently limits the potential for users to develop deep emotional dependencies or misunderstand the AI as a sentient friend.

However, it is important to note that not all classic scales or measurement methods are suitable for technological innovation, just as not all scales are appropriate for adaptation into structured interviews. Moreover, although this study used an AI-based automated assessment method, this approach is still in its early stages and has not fully integrated all possible AI technological applications. As of the drafting of this report, products such as DeepSeek R1, ChatGPT-5, Claude, Grok, and others are rapidly evolving, and there are differences in how systems understand prompts and analyze various languages. While comparing the performance of different models or products was beyond the scope of this study, several other limitations warrant careful consideration. First, the sample was relatively homogeneous, with most participants being young adults living in urban areas and a mixture of university students and local residents. This limits the generalizability of the results to more diverse demographic groups, including older adults or individuals from different socioeconomic and cultural backgrounds. Second, participants with severe depression were excluded from the study. While this decision was made to ensure participant safety and avoid confounding factors such as impaired interaction with the automated system, it also means that the findings may not fully capture the system’s utility in more severe clinical contexts. Completion of the AAP in this study required stable internet and virtual private network access, and several withdrawals were attributable to technical access issues, which are unlikely to be systematically associated with depressive status and, thus, may approximate missingness completely at random. Nonetheless, some selection effects cannot be fully excluded. Third, the interactive assessment relied on 1-language responses and was validated within a single cultural context. Given that LLMs can exhibit variability in comprehension and semantic interpretation across languages and cultural nuances, future studies should examine cross-linguistic and cross-cultural adaptations of this paradigm to ensure robustness and fairness. In addition to these methodological considerations, ethical and practical concerns warrant deeper reflection. Overreliance on AI in mental health assessments carries notable risks, and we emphasize that these tools should complement the clinical judgment of trained professionals rather than attempt to replace it. LLMs can reflect biases from their training data, which may affect how responses are interpreted across different demographic or cultural groups. To ensure that the AAP is used safely and fairly, ethical safeguards, including human oversight, bias monitoring, and ongoing validation, need to be integrated alongside technical improvements.

## Abbreviations

AAP: automated assessment paradigm
AI: artificial intelligence
AUC: area under the curve
BDI-FS: Beck Depression Inventory Fast Screen
BDI-FS-GPT: Beck Depression Inventory Fast Screen via a custom ChatGPT interface
DSM-IV: Diagnostic and Statistical Manual of Mental Disorders, Fourth Edition
LLM: large language model
PHQ-9: Patient Health Questionnaire–9
ROC: receiver operating characteristic
